# Experimental and Numerical Studies on Wave Breaking Characteristics over a Fringing Reef under Monochromatic Wave Conditions

**DOI:** 10.1155/2014/570325

**Published:** 2014-09-03

**Authors:** Jong-In Lee, Sungwon Shin, Young-Taek Kim

**Affiliations:** ^1^Department of Marine and Civil Engineering, Chonnam National University, 50 Daehak-ro, Yeosu, Jeonnam 500-749, Republic of Korea; ^2^Experimental Center for Coastal & Harbor Engineering, Chonnam National University, 50 Daehak-ro, Yeosu, Jeonnam 500-749, Republic of Korea; ^3^River and Coastal Research Division, Korea Institute of Construction Technology, Goyang-si, Gyeonggi-do 411-712, Republic of Korea

## Abstract

Fringing reefs play an important role in protecting the coastal area by inducing wave breaking and wave energy dissipation. However, modeling of wave transformation and energy dissipation on this topography is still difficult due to the unique structure. In the present study, two-dimensional laboratory experiments were conducted to investigate the cross-shore variations of wave transformation, setup, and breaking phenomena over an idealized fringing reef with the 1/40 reef slope and to verify the Boussinesq model under monochromatic wave conditions. One-layer and two-layer model configurations of the Boussinesq model were used to figure out the model capability. Both models predicted well (*r* ^2^ > 0.8) the cross-shore variation of the wave heights, crests, troughs, and setups when the nonlinearity is not too high (*A*_0_/*h*_0_ < 0.07 in this study). However, as the wave nonlinearity and steepness increase, the one-layer model showed problems in prediction and stability due to the error on the vertical profile of fluid velocity. The results in this study revealed that one-layer model is not suitable in the highly nonlinear wave condition over a fringing reef bathymetry. This data set can contribute to the numerical model verification.

## 1. Introduction

Wave breaking in the coastal region is one of the most important hydrodynamic quantities in terms of the beach morphology, sediment transport, wave energy dissipation, and so forth. As waves approach the coast, the wave steepness increases based on the shoaling effect which is controlled by the water depth. In shallow water region, waves start breaking in certain ratio of the wave height to the water depth (Iversen [1]). After waves are broken, the wave heights decrease due to the wave energy dissipation and the broken waves induce longshore currents and wave setup (an increase in the mean water level). The wave breaking also generates turbulence and destabilizes the sea bottom sediment in the surf and swash zone. Therefore, understanding these kinds of physics under the various types of the bottom topography through the field observations and laboratory experiments and modeling the hydrodynamics of wave breaking accurately are very important.

Coral reefs are some kinds of underwater structures settled near islands. One typical type of coral reefs is the fringing reef that consists of the sloping reef face and the mild or almost flat slope from the reef edge to the coastline. Fringing reefs protect the coastal area by inducing wave breaking and wave energy dissipation over the long and wide reef flat area. However, modeling of wave transformation and energy dissipation on this topography is still challenging due to the unique bottom topography.

During past decades, many researchers have studied nearshore wave breaking in terms of wave transformation, energy dissipation, turbulence, and sediment transport through analytical, numerical, and experimental approaches. Thornton and Guza [2] performed field observations and suggested a model for wave height variation based on the energy dissipation model. Smith [3] summarized the nearshore wave breaking and decay research in the surf zone in terms of incipient breaker indices, surf zone wave decay expressions, wave breaking over reefs, and so forth. Cross-shore fluid velocities and wave breaking turbulence in the surf and swash zone were also investigated by many researchers (George et al. [4], Butt and Russell [5], Trowbridge and Elgar [6], Puleo et al. [7], Butt et al. [8], etc.) because the bore generated turbulence affects the sediment transport mechanism. Some laboratory experiments were conducted to understand wave breaking phenomena in both small and large scale wave tanks (Stive [9], Nadaoka and Kondoh [10], Svendsen [11], Nadaoka et al. [12], Cox et al. [13, 14], Ting and Kirby [15, 16], Cox and Kobayashi [17], Petti and Longo [18], Cowen et al. [19], Scott et al. [20], Shin [21], and Shin and Cox [22]). Ting and Kirby [15] conducted laboratory experiments under the different breaker type conditions to estimate the wave breaking turbulence and suggested that the transport of wave breaking turbulence can be correlated to the sediment transport in the surf zone. The modeling of hydrodynamics in the surf and swash zone has been tried by using various numerical schemes. Wurjanto and Kobayashi [23] developed a numerical model (RBREAK2) based on the depth-averaged nonlinear shallow water equations. Raubenheimer [24] used this model to compare the model results to the field data observed at Scripps Beach. Kirby et al. [25] developed a numerical model (FUNWAVE) based on the fully nonlinear Boussinesq equations by extending the Boussinesq equation originally suggested by Peregrine [26]. Since the nonlinear shallow water equations and the Boussinesq equations are depth uniform models, Lynett and Liu [27, 28] derived the higher order Boussinesq equations (COULWAVE) by considering *N* vertical layers (multilayer model) to consider the vertical variation of hydrodynamic quantities. Hsiao et al. [29] used the COULWAVE to simulate wave evolution in a constant deep water depth. They successfully compared their model results with the experimental data in the case of nonlinear monochromatic and bichromatic waves. Lin and Liu [30, 31] and Bradford [32] developed numerical models based on the two-dimensional vertical Reynolds averaged Navies-Stokes equations (RANS, here in after) with a *k* − *ε* turbulence closure scheme. Numerical models with higher complexity were also developed based on the large eddy simulation (Christensen and Deigaard [33]) and direct numerical simulation by other researchers. Shin [21] used four numerical models based on the different governing equations as mentioned above to simulate the wave transformation and turbulence in the surf and swash zone and to verify their results to the experimental results. The numerical results showed that the Boussinesq equations based model was quite creditable to simulate the wave transformation both outside and inside the surf zone in terms of accuracy and computational time expense. However, most of studies including field observations, laboratory experiments, and numerical studies were focused on the wave breaking over plane beaches.

Gourlay [34] conducted laboratory experiments to investigate the wave transformation over a fringing reef with a steep slope and an outer reef-top slope gradually decreasing in the landward direction. Gourlay used a nonlinearity parameter to classify wave transformation regimes whether the breaker type is plunger or spiller. Data was obtained for various wave conditions and water levels. Allsop et al. [35] conducted laboratory experiments of reef type structure by using irregular waves to investigate the wave breaking characteristics under the different wave conditions and the results were compared with predictions for root-mean-squared, significant, and maximum wave heights in terms of wave shoaling and breaking. Su et al. [36] applied a nonlinear wave model based on the mild-slope equation and modified the parametric model to predict wave breaking characteristics on steep reefs. The results were compared with the laboratory experiments conducted by Demirbilek et al. [37] and checked the dependency of the incipient breaker depth index, the breaking-intensity parameter, and the nonlinearity parameter on the measured data. Goertz et al. [38] applied probabilistic bulk dissipation models to estimate wave energy dissipation on a reef structure by comparing the results to the two-dimensional laboratory experimental data under the irregular wave conditions but no one accurately estimated wave energy dissipation by breaking over the reef structure. Yao et al. [39] conducted laboratory experiments of wave breaking over idealized fringing reef under the monochromatic wave conditions to understand the effects of reef-flat submergence and the fore-reef slope on the wave breaking characteristics. The results showed that the relative reef-flat submergence was an important factor to determine the wave breaking parameters.

As mentioned above, many studies on wave transformation and breaking were performed mostly over natural plane beaches through field observations, laboratory experiments, and numerical modeling. A few studies on the wave breaking over fringing reefs have been performed so far but more experimental data sets are still needed under the different condition such as a different fore-slope and wave condition to understand wave breaking characteristics and to verify numerical models.

For this reason, in this study, two-dimensional laboratory experiments were conducted to investigate the characteristics of wave transformation and breaking over a fringing reef with 1/40 fore-reef slope under monochromatic wave conditions. A numerical model based on the multilayer fully nonlinear Boussinesq equations was employed to simulate the wave transformation and breaking over the idealized fringing reef by comparing the results with the experimental data. The second section describes the theoretical background and setup of the numerical model. The third section covers the experimental setup and test conditions and the fourth section shows the results of numerical and experimental results under the different water depth and wave conditions. The last section summarizes the results and ends up to conclusions.

## 2. Numerical Model

So far, researchers have derived Boussinesq equations to apply the wave propagation and transformation in one-dimensional and two-dimensional domain. Depth-integrated equations are the simplified form from three-dimensional problems to two-dimensional problems by applying polynomial approximation to the vertical velocity field. The Boussinesq equations derived by Peregrine [26] and Wu [40] applied the second-order polynomial to the vertical velocity field and have two major problems. First, frequency dispersion in wave propagation is too weak in the intermediate water depth. Second, due to the weakly nonlinear assumption, it is difficult to analyze with higher accuracy when nonlinearity is very high. This limitation comes from the basic assumption that the original equations derived by Peregrine [26] and Wu [40] are weakly nonlinear and dispersive; that is, *O*(*μ*_0_^2^) = *O*(*ε*_0_) ≪ 1, where *μ*_0_ = *kh*, *ε*_0_ = *A*/*h*, and *k*, *h*, and *A* represent the wave number, water depth, and wave amplitude, respectively. Madsen and Sørensen [41] modified the dispersion term and Nwogu [42] improved the frequency dispersion in the previous Boussinesq equations by using fluid velocity in the arbitrary depth. Liu [43] and Wei et al. [44] extended the equations derived by Nwogu [42] to make it applicable in the intermediate depth region. The equations were derived to be able to analyze the propagation of strong nonlinear waves (*ε*_0_ = *O*(1)) and were applicable to the case of *kh* ≈ 3.

Later, while these equations employed second-order polynomial approximation, Gobbi et al. [45] derived the higher-order Boussinesq equations by using fourth-order polynomial so that they improved linear dispersion characteristics up to *kh* ≈ 6. Agnon et al. [46] and Madsen et al. [47] tried similar approach to Gobbi's derivation.

Lynett and Liu [27, 28] derived the Boussinesq equations by dividing the water column with multiple layers (*N* layers), applied the second-order polynomial approximation instead of the higher-order polynomial approximation, and connect the layers each other. Hsiao et al. [29] simulated wave propagation in deep water region on the flat bottom and compared the results to the experimental data. The comparison was successful up to *kA* = 0.0627~0.1577.

In this study, the numerical model developed by Lynett and Liu [26, 27] was used to verify the model by comparison with the experimental results. In order to do that, two-dimensional laboratory experiments were conducted over a fringing reef to investigate the wave transformation and breaking characteristics. The numerical simulations were conducted under the one-layer and two-layer conditions to compare the results between the two-layer model and the extended Boussinesq type model (one-layer model).

### 2.1. Governing Equations

Since the details derivation of *N*-layer Boussinesq equations are described in Lynett and Liu [28], the present study briefly introduces the derived equation applicable to the two-layer condition. Lynett and Liu [28] proved that the two-layer model gave accurate linear characteristics until *kh* is around 8 and nonlinear characteristics until *kh* is around 6. Therefore, the two-layer approach was employed to predict the wave transformation under the wave condition of this study. Two-layer Boussinesq model divides the water column into two layers and integrates the fluid velocities and pressure of the boundary. Equations (1) and (2) are the continuity equation of two layers and the equation of motion for upper layer. Equation (3) is the matching equation of two layers. Consider

(1) ∂ζ∂t+∇·[(ζ−η)u1+(η+h)u2] −∇·{[η3+h36+(η+h)κ222]∇S2     +[η2−h22−(η+h)κ2]∇T2} −∇·{[ζ3−η36+(ζ−η)κ122]∇S1     +[ζ2−η22−(ζ−η)κ1]∇T1},

(2) ∂u1∂t+12∇(u1·u1)+g∇ζ +∂∂t{κ122∇S1+κ1∇T1−∇(ζ22S1)−∇(ζT1)} +∇{∂ζ∂t(T1+ζS1)+(κ1−ζ)(u1·∇)T1   +12(κ12−ζ2)(u1·∇)S1+12(T1+ζS1)2} −Rb+Rf+νT{∇S1−∇2u1        −∇2[κ122∇S1+κ1∇T1]        +∇[ζ22∇2S1+ζ∇2T1]}=0,

(3) u2+κ22−η22∇S2+(κ2−η)∇T2 =u1+κ12−η22∇S1+(κ1−η)∇T1.

**Figure 1:**
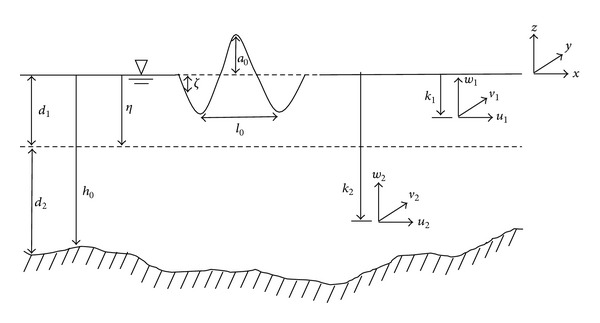
Definition sketch of two-layer model.

As shown in Figure 1, *ζ*, *h*, and *g* denote, respectively, the free surface elevation, the water depth, and the gravitational acceleration. **u**_1_ and **u**_2_ are the fluid velocity vectors of upper and lower layers in two horizontal directions. *R*_*b*_, *R*_*f*_, and *v*_*T*_ in (2) denote energy dissipation terms due to the wave breaking, the bottom friction, and the eddy viscosity, respectively.

In (1) to (3), *S* and *T* are as follows:

(4) $$\begin{array}{c} S_{2}=\nabla \cdot u_{2},\;\;T_{2}=\nabla \cdot \left(hu_{2}\right),\\ S_{1}=\nabla \cdot u_{1},\;\;T_{1}=\eta \left(S_{2}-S_{1}\right)+T_{2}. \end{array}$$

As shown in Figure 1, *d*_1_ and *d*_2_ are characteristic thicknesses and *h*_0_, *a*_0_, and *l*_0_ represent, respectively, a characteristic water depth, a characteristic wave amplitude, and a characteristic wave length. *κ*_1_ and *κ*_2_ are the elevation levels to define **u**_1_ and **u**_2_, which are set to *κ*_1_ = −0.127*h* and *κ*_2_ = −0.618*h* in this study. The boundary level (*η*) between the layers is set to −0.266*h*. When *κ*_1_ is −0.127*h* and *κ*_2_ is ( = −*h*), the governing equation becomes the strong nonlinear Boussinesq equations derived by Liu [43] and Wei et al. [44]. This case is called one-layer model in the present study.

### 2.2. Energy Dissipation Terms

As described in (2), the model considers the energy dissipation due to the wave breaking (*R*_*bx*_ and *R*_*by*_), the bottom friction (*R*_*f*_), and the eddy viscosity (*ν*_*T*_). Equation (5) shows the energy dissipation due to the bottom friction

(5) $$\begin{array}{c} R_{f}=\frac{f}{h+\zeta }u_{b}\left|u_{b}\right|. \end{array}$$

In (5), **u**_**b**_ is the fluid velocity near the bottom, and the bottom friction coefficient (*f*) is set to 0.001.

The energy dissipation due to the wave breaking is shown in (6) which is suggested by Madsen et al. [47]. Consider

(6) $$\begin{array}{c} R_{bx}=\frac{1}{H}\left\{{\left[\nu_{T}{\left(Hu_{1}\right)}_{x}\right]}_{y}+\frac{1}{2}{\left[\nu_{T}{\left(Hu_{1}\right)}_{y}+\nu_{T}{\left(Hv_{1}\right)}_{x}\right]}_{y}\right\},\\ R_{by}=\frac{1}{H}\left\{{\left[\nu_{T}{\left(Hv_{1}\right)}_{y}\right]}_{y}+\frac{1}{2}{\left[\nu_{T}{\left(Hu_{1}\right)}_{y}+\nu_{T}{\left(Hv_{1}\right)}_{x}\right]}_{x}\right\}. \end{array}$$

In (6), *H* is total water depth and the eddy viscosity is described as

(7) $$\begin{array}{c} \nu_{T}=BH\zeta_{t}. \end{array}$$

*B* is defined as a parameter to categorizing breaking and nonbreaking condition in

(8) $$\begin{array}{c} B=\{\begin{array}{cc} \delta , & \zeta_{t}\geq 2\zeta_{t}^{b},\\ \delta \left(\frac{\zeta_{t}}{\zeta_{t}^{b}}-1\right), & {\zeta_{t}^{b}\leq \zeta }_{t}\leq 2\zeta_{t}^{b},\\ 0, & \zeta_{t}\leq \zeta_{t}^{b}, \end{array} \end{array}$$

where the amplification factor (*δ*) is set to 6.5 and *ζ*_*t*_^*b*^ is the parameter identifying continuing and stopping the wave breaking defined in

(9) $$\begin{array}{c} \zeta_{t}^{b}=\{\begin{array}{cc} \zeta_{t}^{\left(F\right)}, & t-t_{0}\geq T^{b},\\ \zeta_{t}^{\left(I\right)}+\frac{t-t_{0}}{T^{b}}\left(\zeta_{t}^{\left(F\right)}-\zeta_{t}^{\left(I\right)}\right), & 0\leq t-t_{0}<T^{b}. \end{array} \end{array}$$

In (9), *t*_0_ is the starting time of the wave breaking. The other parameters in (8) and (9) are defined as $\zeta_{t}^{\left(I\right)}=0.65\sqrt{gH}$, $\zeta_{t}^{\left(F\right)}=0.08\sqrt{gH}$, and $T^{b}=8.0\sqrt{H/g}$ (Lynett et al. [48]). The superscripts *I* and *F* represent “initial” and “final.”

## 3. Experimental Setup

**Figure 2:**
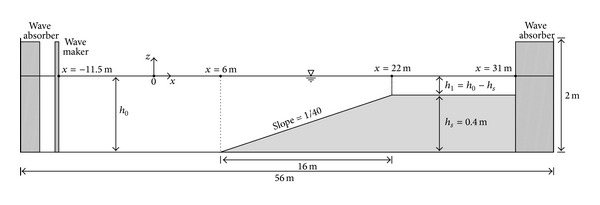
Experimental and numerical model setups.

Two-dimensional laboratory experiments were conducted in a glass-walled wave flume in Korea Institute of Construction Technology (KICT) as shown in Figure 2. The flume with the length of 56.0 m, width of 1.0 m, and height of 2.0 m was used to investigate the wave transformation and breaking phenomena on the idealized fringing reef. A piston type wave generator was equipped in the flume, which is capable of generating regular and irregular waves with various spectral shapes (JONSWAP, TMA, Bretschneider-Mitsuyasu, etc.). Wave absorbers were installed in both side of the flume to reduce the reflected waves. The active wave absorption system was also used to control wave amplification by reflected wave and it uses the water surface elevation data collected by a wave gauge mounted on the wave generator. A 1/40 beach slope was installed in the flume and the flat section was followed by the end of the slope to reproduce the idealized fringing reef.

Figure 2 shows a schematic view of experimental and numerical modeling setups. In the numerical model domain point of view, the wave generator in the flume was located at *x* = −11.5 m. The beach slope started at *x* = 6 m and ended at *x* = 22 m so that the height of reef flat (*h*_*s*_) was set to 0.4 m. Capacitance type wave gauges were deployed every 0.5 m from the beginning of the slope to the wave breaking region and every 0.25 m near the wave breaking region. The data were collected through the data acquisition system with the sampling rate of 20 Hz.

A total of 12 different wave conditions with two different water depths were tested to investigate the wave transformation and breaking phenomena over a fringing reef through the laboratory experiments and numerical modeling. In Table 1, *T*_0_, *A*_0_, and *k*_0_ represent the incident wave period, wave amplitude, and wave number, respectively. The subscript 0 represents the incident wave condition at *x* = 0 m. The wave period varied from 1.3 to 2.2 sec with the increment of 0.3 sec and the incident wave height was set to 0.04, 0.08, and 0.12 m.

In the laboratory experiments, monochromatic waves were generated for 175 sec and the data were used between 60.05 and 162.40 seconds to calculate the wave characteristic quantities, for example, wave heights, wave crest and trough, and mean water level. Each wave condition was repeated three times in order to reduce measurement errors. In numerical model domain, the model set the grid spacing (Δ*x*) to *λ*_0_/50 and the time step (Δ*t*) based on the Courant number ($\Delta t\sqrt{gh}/\Delta x$) of 0.5 as mentioned in Peregrine [26], where *λ*_0_ represents wave length at *x* = 0 m.

## 4. Results and Discussion

**Figure 3:**
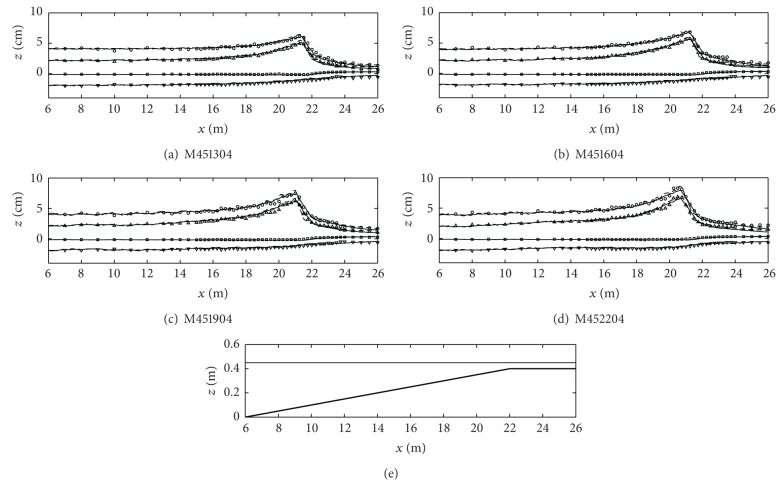
Cross-shore variations of the wave heights (circles), the wave crest levels (triangles), the wave trough levels (inverse triangles), and the mean water level (squares). All symbols are the experimental results and lines are the numerical model results (one-layer model: dashed lines; two-layer model: solid lines). The bottom figure is the fringing reef profile and the free surface level (case ID: M451304 through M452204).

Figure 3 shows the numerical and experimental results (case ID: M451304 through M452204) of the cross-shore variations of the wave heights (circles), the wave crest and trough levels (triangles and inverse triangles), and the mean water levels (squares) under the different wave periods when the water depth (*h*_0_) is 0.45 m at the offshore boundary. As shown in Table 1, the nonlinearity (*A*_0_/*h*_0_) of this case is smaller than other cases. In all four figures, numerical model results of both one-layer (dashed lines) and two-layer (solid lines) models qualitatively agree with the experimental results in terms of the wave transformation, breaking, and setup.

**Figure 4:**
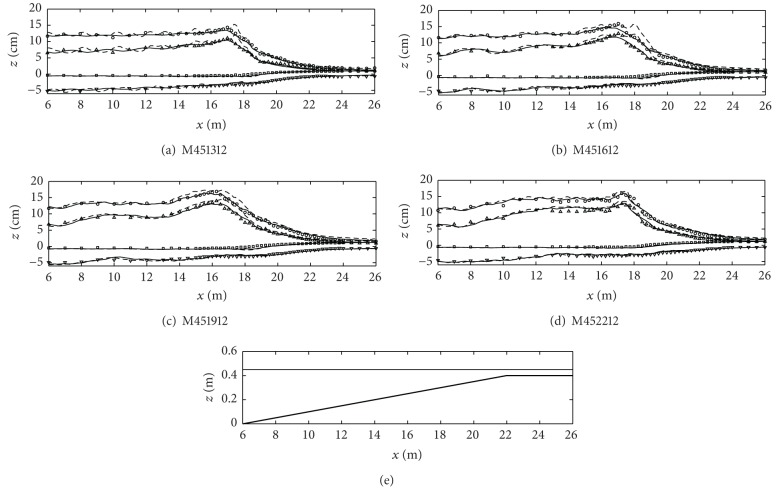
Cross-shore variations of the wave heights (circles), the wave crest levels (triangles), the wave trough levels (inverse triangles), and the mean water level (squares). All symbols are the experimental results and lines are the numerical model results (one-layer model: dashed lines; two-layer model: solid lines). The bottom figure is the fringing reef profile and the free surface level (case ID: M451312 through M452212).

Figure 4 shows the results at the same water depth (case ID: M451312 through M452212) as Figure 3 but the nonlinearity is higher as shown in Table 1. The results in Figure 4 show that the one-layer model predicts less accurately compared to the two-layer model's prediction. In terms of wave steepness (*k*_0_ *A*_0_), the case in Figure 4(a) has the highest wave steepness. As the wave steepness increases, the one-layer model poorly predicts the wave heights and shows unstable results (fluctuation), especially in the wave breaking region. This may result from the estimate error in the vertical profile of the fluid velocities as the wave goes to the breaking location due to the wave shoaling and the increase in nonlinearity. Lynett and Liu [28] showed the estimate results of the vertical profiles of the horizontal and vertical velocities from the one-layer model to the four-layer model according to the different *kh* values. They concluded that the two-layer model successfully estimated the velocity profiles compared to the linear wave theory while the one-layer model generates large errors in both the horizontal and the vertical velocity profiles. Therefore, one-layer model cannot accurately reproduce the wave transformation and breaking phenomena in this bottom profile under the highly nonlinear wave condition. On the contrary, two-layer model predicts the wave height variation better because the faster horizontal fluid velocity can be well reproduced.

**Figure 5:**
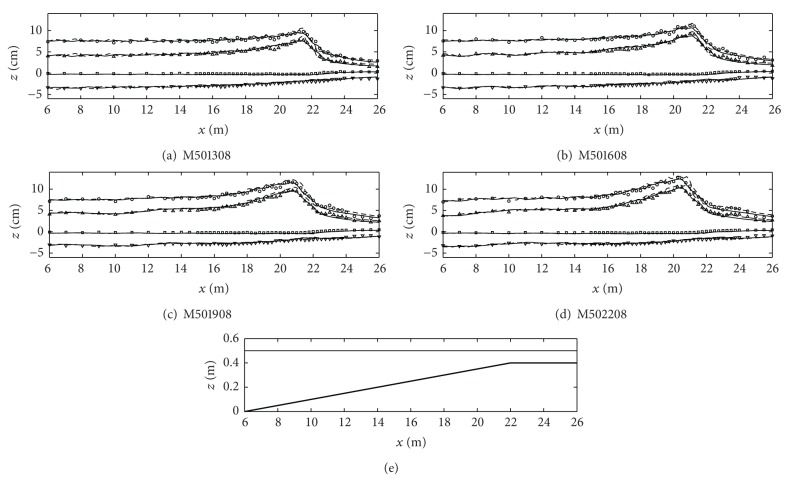
Cross-shore variations of the wave heights (circles), the wave crest levels (triangles), the wave trough levels (inverse triangles), and the mean water level (squares). All symbols are the experimental results and lines are the numerical model results (one-layer model: dashed lines; two-layer model: solid lines). The bottom figure is the fringing reef profile and the free surface level (case ID: M501308 through M502208).

Figure 5 shows the numerical and experimental results (case ID: M501308 through M502208) when the water depth (*h*_0_) is 0.5 m at the offshore boundary and is 0.1 m at the reef flat (*h*_1_). In this figure, even if the present study did not show the results of M451308 to M452208 because of redundancy, the wave breaking location is shifted toward the reef edge compared to the water depth of 0.45 m due to the characteristics of depth-limited breaking. The numerical simulation results from both one-layer and two-layer models predict well the wave transformation, breaking, and setup but the one-layer model results show fluctuations in the wave height and crest predictions a little bit.

**Figure 6:**
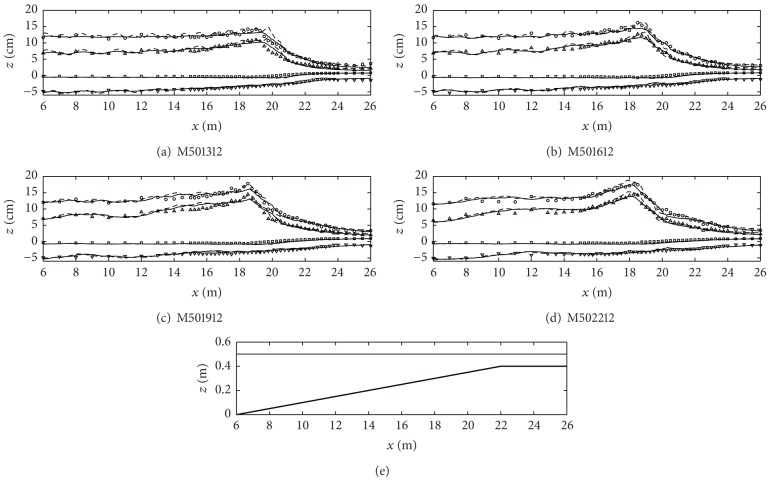
Cross-shore variations of the wave heights (circles), the wave crest levels (triangles), the wave trough levels (inverse triangles), and the mean water level (squares). All symbols are the experimental results and lines are the numerical model results (one-layer model: dashed lines; two-layer model: solid lines). The bottom figure is the fringing reef profile and the free surface level (case ID: M501312 through M502212).

In Figure 6 under the highly nonlinear wave condition with the offshore water depth of 0.5 m, the instability in one-layer model increases as the wave steepness increases. In other words, the fluctuation in the cross-shore variation in wave heights, crests, and troughs appears more seriously in Figure 6(a) and the prediction of wave breaking location is not accurate. However, two-layer model predicts the wave transformation and breaking relatively better.

**Figure 7:**
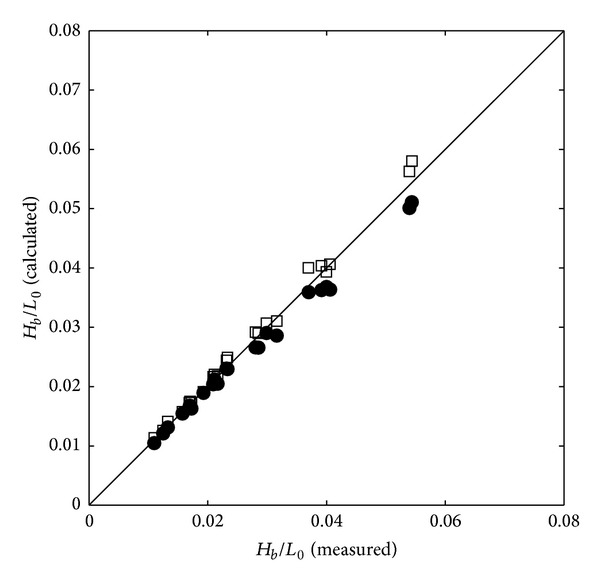
Calculated wave steepness (one-layer model: squares; two-layer model: filled circles) versus measured wave steepness.

Figure 7 shows the comparison of measured and predicted wave steepness using breaking wave heights (*H*_*b*_/*L*_0_) which is commonly used to calculate the surf similarity parameter ($\xi_{b}=\tan\alpha /\sqrt{H_{b}/L_{0}}$) suggested by Madsen et al. [47] where *α* is the beach slope and *H*_*b*_ is the breaking wave height. In this figure, both one-layer (open squares) and two-layer (filled circles) models seem to predict the wave steepness accurately.

**Figure 8:**
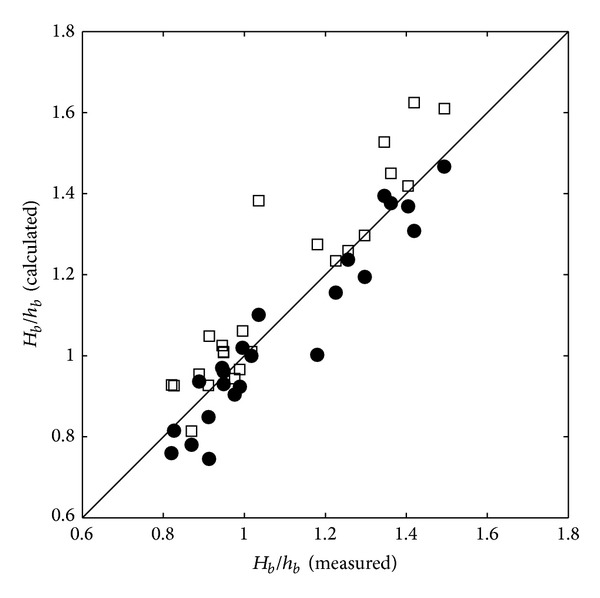
Calculated incipient breaker depth indices (one-layer model: squares; two-layer model: filled circles) versus measured incipient breaker depth indices.

However, as shown in Figure 8, the prediction of the incipient breaker depth index (*H*_*b*_/*h*_*b*_) shows difference in the results from the one-layer and two-layer models. The two-layer model predicts the incipient breaker indices somehow better than the one-layer model does. This is because the two-layer model shows better performance in predicting the breaking wave heights and locations even in the highly nonlinear wave condition as shown in Figures 4 and 6.

**Figure 9:**
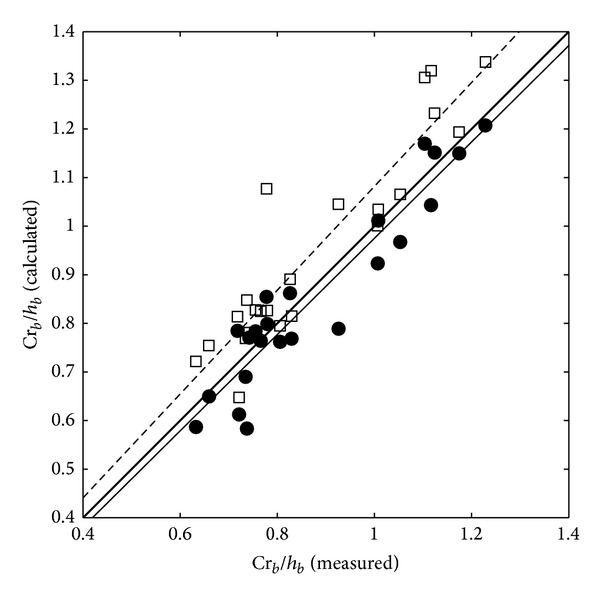
Calculated ratio of the crest level to the breaking water depth (one-layer model: squares; two-layer model: filled circles) versus ratio of the crest level to the breaking water depth. The dashed line and thin solid line are the best fit lines from one-layer and two-layer models, respectively.

In order to investigate the prediction capabilities of the one-layer and two-layer models on the breaking wave heights and the cross-shore location of the wave breaking including the nonlinearity effects, the numerical and experimental results are compared with each other in terms of the wave crest and trough levels.  Figure 9 shows the results of the ratio of the breaking wave crest level (Cr_*b*_) to the wave breaking water depth (*h*_*b*_) in comparison with the numerical model and experimental results. In this figure, the wave breaking water depth is used because it includes the information of the cross-shore location of the wave breaking. The comparison results show similar tendency to the results in Figure 7. The best fit line from the two-layer model results (thin solid line) shows higher correlation with the experimental results than those from the one-layer model (dashed line). Especially, the one-layer model overpredicts the breaking wave crest heights up to 1.4 times. According to the results in Figures 4 and 6, the maximum discrepancy of the prediction may occur in the higher wave steepness and nonlinearity.

**Figure 10:**
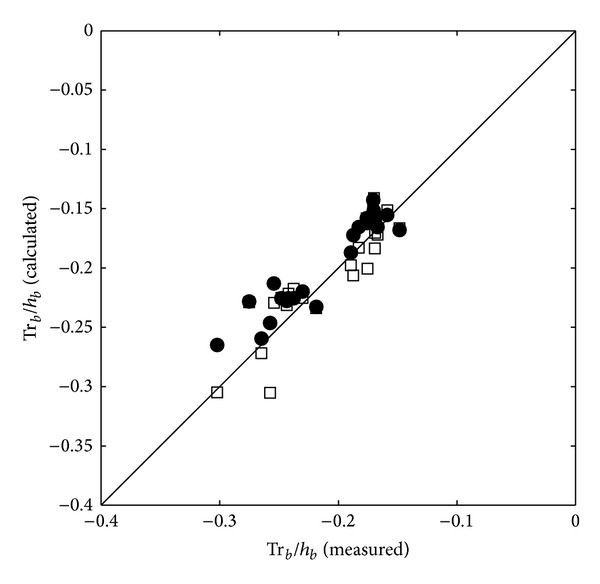
Calculated ratio of the trough level to the breaking water depth (one-layer model: squares; two-layer model: filled circles) versus ratio of the trough level to the breaking water depth.

Figure 10 shows the comparison results of the ratio of the breaking wave trough level (*Tr*⁡_*b*_) to the breaking water depth. In contrast to Figure 9, Figure 10 reveals that both models fairly agree with the experimental results even if the two-layer model slightly overestimates the values while the one-layer model slightly underestimates them. These results prove that the difference in between the predictions of the crest level by the one-layer model and by the two-layer model as the wave nonlinearity and steepness increase. Therefore, two-layer model by defining the horizontal fluid velocity in upper layer is more stable to simulate wave transformation and breaking phenomena in this kind of reef structure even in the higher nonlinear wave condition.

Table 2 shows the statistical values for the numerical model validation including *r* ^2^ values, root-mean-squared errors, and the coefficients of best fit lines. As shown in Table 2, the two-layer model shows higher correlation than the one-layer model. This table also shows that the major difference in between the one-layer and two-layer model results appears in the Cr_*b*_/*h*_*b*_ as shown in Figure 9.

## 5. Conclusions

In the present study, two-dimensional laboratory experiments were conducted to investigate the cross-shore variations of wave transformation, setup, and breaking phenomena over an idealized fringing reef with the 1/40 reef slope and to verify the Boussinesq model. Monochromatic waves were generated in 12 different wave conditions with two different water depths. One-layer and two-layer model configurations of the Boussinesq model were used to figure out the model capability.

The following conclusions are drawn based on the experimental and numerical results in the present study.

1. Both one-layer and two layer models predicted the cross-shore variation of the wave heights, crests, troughs, and setups when the nonlinearity is not too high. However, as the wave nonlinearity and steepness increase, the one-layer model showed problems in prediction and stability due to the error on the vertical profile of the fluid velocity.
2. Both models accurately simulated the wave steepness (*H*_*b*_/*L*_0_) compared with the experimental results. However, in the simulation results of the one-layer model, the fluctuation of the cross-shore wave height variation due to the model instability increases when the nonlinearity and the wave steepness are high.
3. In terms of the incipient breaker depth index (*H*_*b*_/*h*_*b*_), one-layer model showed problems with the prediction while two-layer model results fairly agreed with the experimental results. The difference of the prediction results between one-layer and two-layer models was shown in the crest level rather than the trough level. Therefore, one-layer model is not suitable in the highly nonlinear wave condition over a fringing reef structure.

All the experimental data set has been collected from more than 50 cross-shore locations simultaneously in this study. Therefore, this synoptic data set can contribute to the numerical model verification for numerical model developers.

## Figures and Tables

**Table 1:** Input wave conditions.

Case ID	*T*_0_	*A*_0_/*h*_0_	*k*_0_ *A*_0_	*k*_0_ *h*_0_	*h*_0_	*h*_1_
	(sec)	(—)	(—)	(—)	(m)	(m)
M451304	1.3	0.045	0.057	1.260	0.45	0.05
M451604	1.6	0.045	0.043	0.954		
M451904	1.9	0.044	0.034	0.774		
M452204	2.2	0.044	0.029	0.653		
M451308	1.3	0.090	0.113	1.260		
M451608	1.6	0.084	0.080	0.954		
M451908	1.9	0.086	0.067	0.774		
M452208	2.2	0.083	0.054	0.653		
M451312	1.3	0.133	0.168	1.260		
M451612	1.6	0.134	0.128	0.954		
M451912	1.9	0.135	0.104	0.774		
M452212	2.2	0.128	0.084	0.653		

M501304	1.3	0.039	0.054	1.360	0.50	0.10
M501604	1.6	0.040	0.040	1.021		
M501904	1.9	0.038	0.031	0.824		
M502204	2.2	0.040	0.028	0.693		
M501308	1.3	0.080	0.109	1.360		
M501608	1.6	0.078	0.079	1.021		
M501908	1.9	0.075	0.062	0.824		
M502208	2.2	0.073	0.051	0.693		
M501312	1.3	0.120	0.163	1.360		
M501612	1.6	0.121	0.123	1.021		
M501912	1.9	0.121	0.100	0.824		
M502212	2.2	0.118	0.082	0.693		

**Table 2:** Statistical quantities for the numerical model validation (*r* ^2^ values, root-mean-squared errors (RMSE), and best fit line coefficients).

	One-layer model				Two-layer model			
	*r* ^2^	RMSE	Best fit (*y* = *ax* + *b*)		*r* ^2^	RMSE	Best fit (*y* = *ax* + *b*)	
			*a*	*b*			*a*	*b*
*H*_*b*_/*L*_*b*_	0.995	0.001	1.046	0.000	0.996	0.002	0.901	0.001
*H*_*b*_/*h*_*b*_	0.869	0.113	1.069	−0.005	0.913	0.074	0.986	−0.023
Cr_*b*_/*h*_*b*_	0.851	0.109	1.068	0.014	0.886	0.067	0.991	−0.015
*Tr*⁡_*b*_/*h*_*b*_	0.788	0.021	0.906	−0.017	0.886	0.021	0.821	−0.024
